# The reproducibility of protocols used to mediate a current-induced vasodilation in the human cutaneous microcirculation

**DOI:** 10.1371/journal.pone.0314430

**Published:** 2024-11-27

**Authors:** Alicia Guigui, Léa Liaigre, Matthieu Roustit, Jordan Loader

**Affiliations:** 1 Univ. Grenoble Alpes, Inserm U1300 –HP2, CHU Grenoble Alpes, Grenoble, France; 2 Univ. Grenoble Alpes, Inserm CIC1406, CHU Grenoble Alpes, Grenoble, France; 3 Department of Medical Sciences, Uppsala University, Uppsala, Sweden; Memorial Sloan Kettering Cancer Center, UNITED STATES OF AMERICA

## Abstract

**Introduction:**

Current-induced vasodilation (CIV) can be used to assess the prostacyclin (PGI_2_) pathway. This study, for the first time, evaluated the reproducibility of several protocols used to mediate a CIV.

**Methods:**

Three CIV protocols were evaluated in 10 healthy participants who completed four testing sessions. Two testing sessions were conducted on the calf, separated by a period of seven days allowing interday reproducibility to be assessed. Two testing sessions were also conducted seven days apart on the forearm. At each testing session, cutaneous microvascular assessments were conducted for one hour on the right limb of interest before assessments were immediately performed on the left limb, allowing for intersite, intraday reproducibility to be evaluated. Assessments were then repeated at the same site on the right limb, allowing for intrasite, intraday reproducibility to be evaluated. Reproducibility was assessed using the within-subject coefficients of variation and the intra-class correlation coefficients.

**Results:**

Protocol A (Pulses of 0.03, 0.06, 0.09, 0.12, 0.15, and 0.18 mA for 10 s each; 60 s intervals), Protocol B (0.1 mA for 60 s), and Protocol C (2 pulses of 0.1 mA for 10s each; 240 s interval) had good to excellent interday reproducibility for calf and forearm assessments. The intrasite, intraday reproducibility of each protocol was less clear. Intersite testing didn’t improve intraday reproducibility. Reproducibility was consistently unacceptable when the microvascular response to the electrical stimulation was expressed as the absolute change and the percentage change between baseline values and the maximal plateau. A microvascular response wasn’t induced ∼10% of assessments on either the calf or forearm.

**Conclusions:**

This study indicates that a CIV is most reproducible with interday testing and when data are expressed as the maximal plateau in perfusion units or as cutaneous vascular conductance, and as the area under the curve.

## Introduction

It is well accepted that impaired microvascular reactivity precedes and contributes to the development of obesity, type 2 diabetes and diabetes-related complications [1]. Noting that impairments in microvascular reactivity can be caused by disruptions to multiple physiological pathways [2], the prostacyclin (PGI_2_) pathway has rarely been focused on in *in vivo* studies of human microvascular function. The cutaneous PGI_2_ pathway and its capacity to modulate a microvascular response (i.e., vasodilation) can be studied using non-invasive reactivity tests.

Previous work has shown that the PGI_2_ pathway can be challenged *in vivo* in the cutaneous microcirculation by delivering a low intensity electrical current to the skin surface. The resulting vasodilation, often referred to as current-induced vasodilation, has been shown to be axon reflex- and cyclooxygenases (COX)-dependent, as it is inhibited by capsaicin and aspirin [3–7]. Experimental studies have further shown that vasodilation induced by a cathodal current mainly relies on the release of PGI_2_ produced through the COX-1/PGIS pathway [8]. A clinical study, using celecoxib as a preferential COX-2 inhibitor, confirmed this hypothesis as it did not alter cutaneous current-induced vasodilation [9].

Several different protocols that mediate a current-induced vasodilation have been published [3–5, 7, 10, 11]. However, no study has determined if the microvascular responses induced by any of these protocols are reproducible. Additionally, to our best knowledge, majority of published human data only details the use of these protocols on the forearm. While it’s suggested that the cutaneous microcirculation at the forearm might provide a site that is representative of the systemic microvascular network, it remains advantageous if microvascular reactivity can be assessed on or close to the site where vascular-related complications occur (e.g., on the lower leg, near to where diabetic foot ulcers form). Considering this, the aim of this study was to evaluate, both on the forearm and the calf, the intraday (intrasite and intersite) and interday reproducibility of three different published protocols that mediate a current-induced vasodilation.

## Materials and methods

### Study population

Ten healthy volunteers >18 years of age were recruited at the Centre Hospitalier Universitaire de Grenoble, France. Potential participants were excluded if they had: 1) any significant medical history (e.g., renal disease, cardiovascular disease, diabetes, epilepsy); 2) any injury or scarring to the skin sites on which assessments would be conducted; 3) if they had used nonsteroidal anti-inflammatory drugs within seven days of their participation in this study; or 4) if they had a history of habitual cigarette use. A pregnancy test was performed at inclusion for women. Women who could become pregnant were required to be using an effective contraception. The protocol of this study was approved by the institutional review board at the Centre Hospitalier Universitaire de Grenoble, France, on January 7^th^, 2022 (Approval reference: ID-RCB 2020-A02191-38). Each participant provided written informed consent before entering the protocol.

### Study design

All clinical and vascular assessments were performed in a temperature-controlled room (23°C ± 1°C) at the Grenoble Clinical Research Center, Centre Hospitalier Universitaire de Grenoble, France, between February 16, 2022, and January 25, 2023. To standardize the preparation for each vascular assessment and limit factors that may influence vascular reactivity, the participants were instructed to abstain from strenuous exercise for the 24 hours, alcohol consumption for the 12 hours, and caffeine consumption for the 12 hours preceding each testing session. Additionally, to minimize postprandial variations in vascular reactivity, participants were required to present to each testing session in a fasted state (i.e., at least eight hours fasted). Participants were also instructed to maintain their normal level of physical of activity in the lead-up to and throughout their participation in the study protocol.

To evaluate the reproducibility of the three protocols used to mediate a current-induced vasodilation, participants completed four testing sessions; inclusive of two testing sessions where cutaneous microvascular assessments were conducted on the calf, each separated by a period of seven days; and another two testing sessions, also conducted seven days apart, where assessments were performed on the forearm. At the beginning of each testing session, participants entered an acclimatization period in which they remained laying in a supine position for a minimum of 20 minutes. During this time, three adhesive electrodes that deliver the electrical current were positioned on each calf or each forearm.

**Fig 1:**
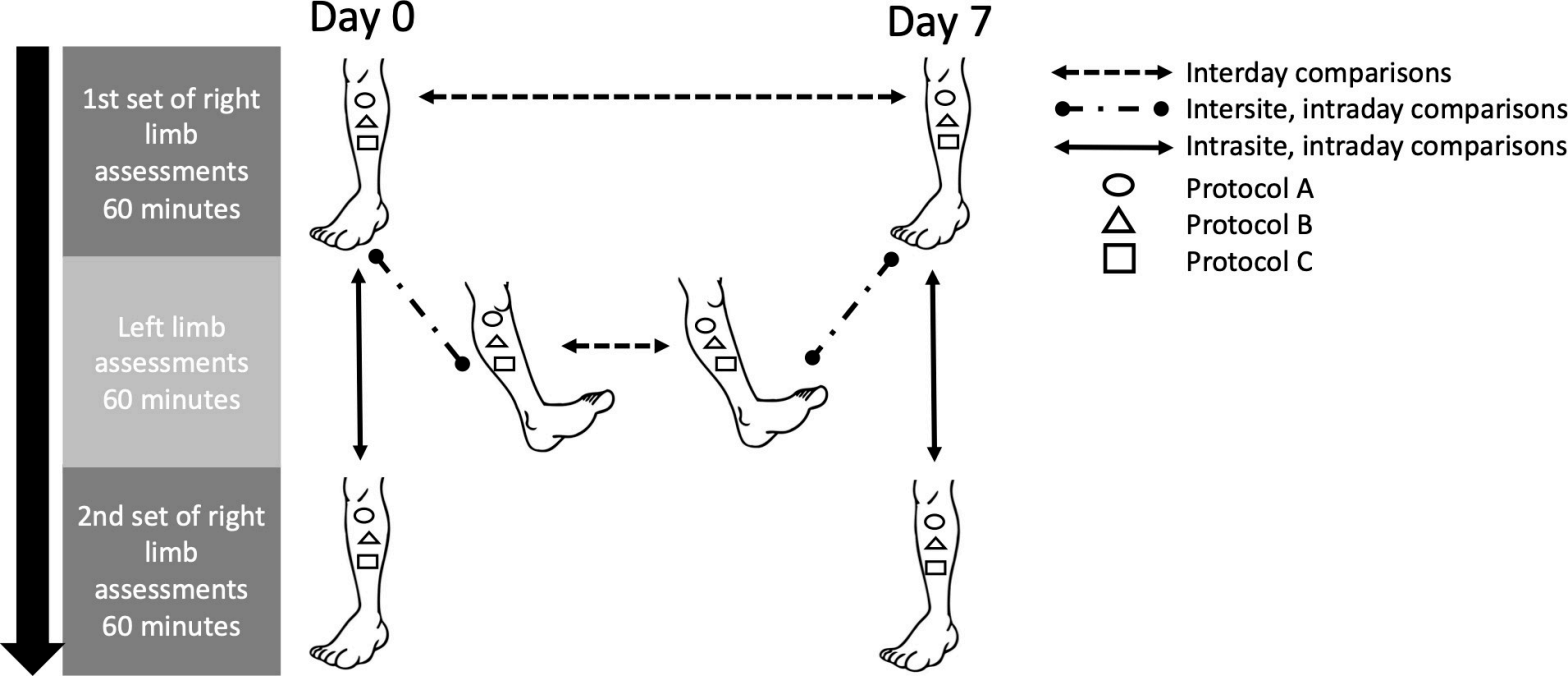
The sequence of assessments at each testing session and the available comparisons in this study. Each arrow represents 10 possible comparisons. Thus, 20 comparisons were available to determine the 1) intrasite, intraday reproducibility, 2) intersite, intraday reproducibility, and 3) interday reproducibility of each protocol for mediating a current-induced vasodilation on the calf and the forearm. The electrode (i.e., the distal, middle, or proximal electrode) at which Protocol A, B, and C were tested was selected by randomization for each participant, but those locations remained the same for a given participant in all assessments thereafter.

The first of three rounds of microvascular assessments began immediately after the acclimatization period ended. Before each round, blood pressure and heart rate were measured. Cutaneous microvascular assessments were conducted for one hour on the right limb of interest before assessments were immediately performed for one hour on the left limb. Assessments were then repeated for one hour at the exact same site on the right limb, allowing for intrasite, intraday reproducibility to be assessed. Evaluations of the left limb allowed intersite, intraday reproducibility to be assessed. That is, to determine if intraday reproducibility is influenced by same-site assessments in a single testing session or if it is improved by changing sites. While 10 participants were included in the study, a sample of 20 data points (two per participant) were pooled for each outcome for both the calf and forearm: 1) intrasite, intraday reproducibility; 2) intersite, intraday reproducibility; and 3) interday reproducibility (Fig 1).

The adhesive electrodes placed on the right limb during the acclimatization period remained on the same anatomical location that they were initially placed for the duration of the testing session. For assessments of the forearm, participants remained laying in a supine position throughout the entire testing session, from the beginning of the acclimatization period, through to the end of the third round of microvascular assessments. However, for assessments of the calf, participants remained in a reclined, seated position. Resting blood pressure, resting heart rate and capillary blood glucose were measured immediately before the beginning of each round of microvascular assessments. The same sequence of assessments was repeated in all four testing sessions. Each participant’s age, height and weight were recorded at the beginning of the first testing session.

### Clinical measurements

Fasting capillary blood glucose concentrations were assessed using a handheld blood glucose monitoring system (Accu-Chek® Guide, Roche, Mannheim, Germany). Resting blood pressure and heart rate were measured using a digital sphygmomanometer (Infinity gamma XLNI monitor, Draeger Medical Systems, Danvers, USA). Systolic and diastolic blood pressure values were used to calculate mean arterial pressure according to the formula: [(2 x diastolic blood pressure) + systolic blood pressure]/3.

### Cutaneous microvascular assessments

**Fig 2:**
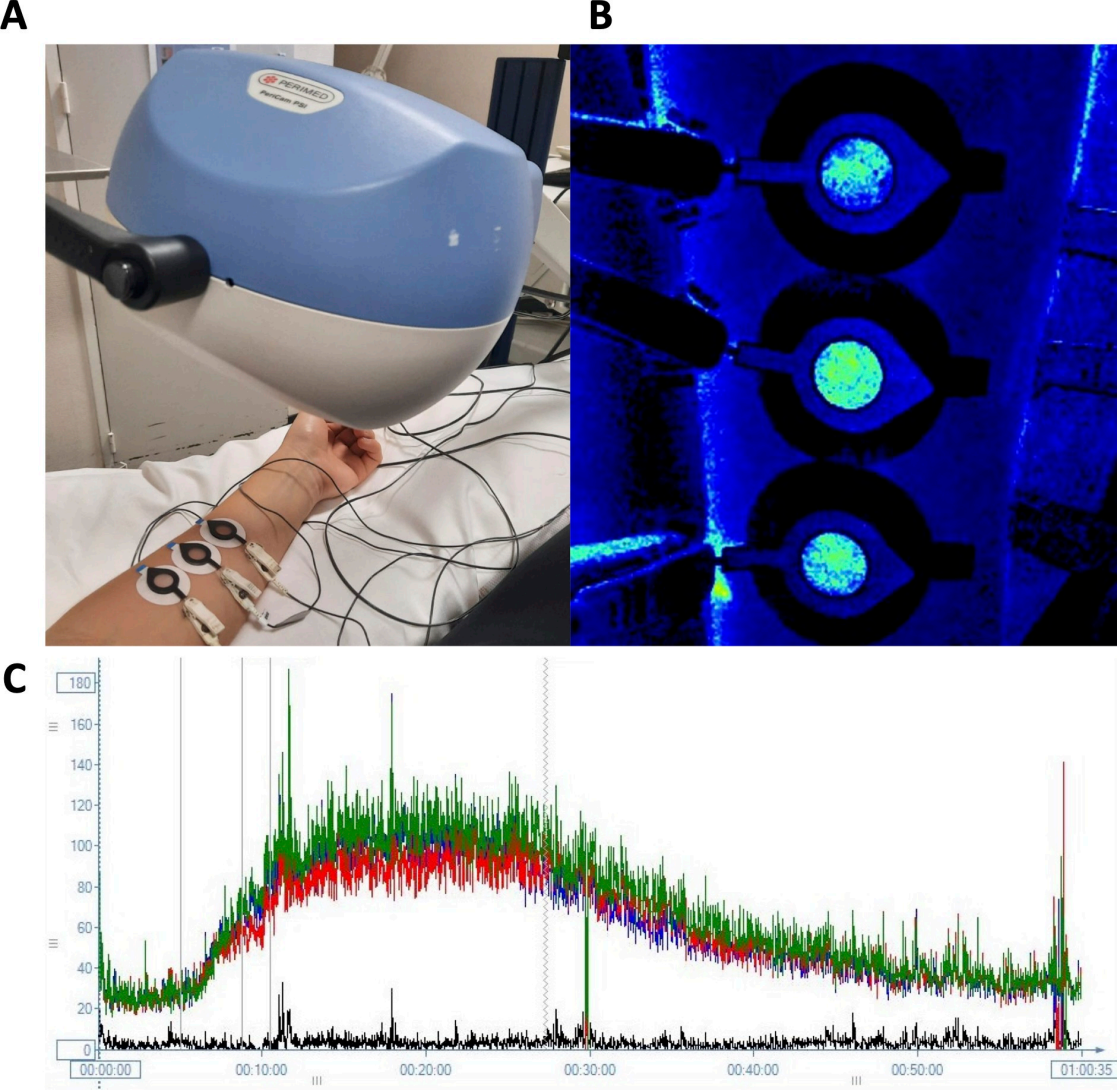
**A)** Placement of the active and dispersive electrodes on the forearm; and **B)** the cutaneous microvascular response to the three protocols used to mediate a current-induced vasodilation. Brighter colours (e.g., light green, yellow, red) within the electrode indicate an increase in blood perfusion compared to darker regions outside the electrode. **C)** The microvascular response in perfusion units as displayed by the Perimed software. Larger spikes indicate artifacts (e.g., movements by the participants) during the recordings, which were retrospectively removed during data-analysis.

The three protocols that were assessed in this study are detailed in Table 1 [7, 10, 11]. Measurements were performed by two researchers. To deliver the electrical current specified by these protocols, a total of six active electrodes (LI 611, Perimed, Järfälla, Sweden) were used in each of the four testing sessions. Three electrodes were placed on the ventral surfaces of both the left and right upper forearms, in assessments of reproducibility on the forearm; and on the medial belly of each gastrocnemius, in evaluations on the calf. The chamber of each active electrode was separated by at least 1 cm and was positioned to avoid hair, broken or irritated skin, areas of increased skin pigmentation, and visible veins. If necessary, hair was removed by shaving at least 24 hours before a testing session began. To complete the electrical circuits, a dispersive electrode (PF 384, Perimed, Järfälla, Sweden) was positioned 10 cm from the chamber of each active electrode (Fig 2). The chambers of the active electrodes were filled with deionized water immediately before measurements of resting (baseline) cutaneous blood perfusion began.

**Table 1:** Protocols for mediating a current-induced vasodilation that were evaluated in this study.

Protocol	Electrical current intensity and duration	Current density (mA/cm²)	Total charge density (mC/cm²)
**A** [10]	Pulses of 0.03, 0.06, 0.09, 0.12, 0.15, and 0.18 mA for 10 s each; 60 s intervals	0.02, 0.04, 0.06, 0.08, 0.10, and 0.12	4.09
**B** [7]	0.1 mA for 60 s	0.06	3.90
**C** [11]	2 pulses of 0.1 mA for 10s each; 240 s interval	0.06	1.30

Each protocol used a cathodal current and deionized water as the conductor.

Changes in cutaneous microvascular blood perfusion, in response to each of the protocols, were quantified with laser speckle contrast imaging, using a 70-mW system with a laser wavelength of 785 nm (PeriCam PSI System^®^, Perimed, Järfälla, Sweden). The working distance of the laser head was 15 cm, the laser measurement area was 20 cm X 20 cm, and the image acquisition rate was one image per second. Images were recorded at a frequency of 18 Hz using a computer with data acquisition software (PimSoft 1.2.2.0^®^, Perimed, Järfälla, Sweden). Resting (baseline) cutaneous microvascular blood perfusion was measured for four minutes before the electrical currents specified in each protocol were delivered to the active electrodes using a USB power supply (PF 751 PeriIont Systems, Perimed, Järfälla, Sweden). The three protocols were tested simultaneously across the three active electrodes, one protocol per electrode. The location (i.e., the distal, middle, or proximal electrode) at which each protocol was tested was selected by randomization for each participant, but those locations remained the same for a given participant in all assessments thereafter. Measurement of cutaneous microvascular blood perfusion continued uninterrupted for one hour from the beginning of the baseline measurement.

### Data analysis

The PimSoft data acquisition software was used to set circular regions of interest that covered the entire chamber window for each active electrode. These regions of interest were repositioned retrospectively to ensure that they remained over the chamber windows for the duration of the laser speckle recordings, adjusting for any movements made by the participant during the assessments. This part was done by one researcher and validated by a second one. All data were exported from PimSoft to Microsoft Excel and coded by a researcher not involved in data collection or analyses, to blind the investigator.

Cutaneous microvascular perfusion values were averaged for the two minutes immediately prior to the beginning of electrical current delivery (baseline data) and for the two minutes at the maximal plateau in blood perfusion thereafter. The cutaneous microvascular response to each protocol was expressed as the maximal plateau in blood perfusion, the change in blood perfusion between baseline and maximal plateau values, the percentage increase in blood perfusion from baseline to maximal plateau values, and the area under the curve [10]. Where applicable, data were expressed as perfusion units and cutaneous vascular conductance, which is perfusion units divided by mean arterial pressure; accounting for variations in blood pressure between each round of microvascular assessments [10]. In any instance where there was a reduction in blood perfusion from baseline measurements following electrical stimulation, values were imputed to indicate a positive yet minimal vascular response (e.g., an increase of 1% from baseline values) to allow the subsequent statistical analyses to be conducted.

### Statistical analysis

The intraday and interday reproducibility of each protocol were assessed using the within-subject coefficients of variation with <35% deemed acceptable; and the intra-class correlation coefficients with values of <0.40, 0.40 to 0.75 and >0.75 representing poor, fair to good, and excellent agreements, respectively [10, 12–18]. Descriptive data and laser speckle contrast imaging data were presented as mean ± standard deviation. Statistical comparisons were performed using Microsoft Excel, SPSS (version 29; IBM Corp., Armonk, NY, USA), and Jamovi v2.3.21.0.

## Results

### Study population

Ten healthy participants (age, 29.70 ± 6.96 years; body mass index, 22.30 ± 2.65 kg/m^2^), including six women, completed the study protocol. Contraceptive methods among the six women included contraceptive pill (n = 3), implant (n = 1), copper coil (n = 1), and abstinence (n = 1). Resting heart rate, and resting blood pressure were similar between each testing session (Table 2). However, fasting blood glucose concentrations were different between day one and day seven forearm assessments.

**Table 2:** Resting clinical characteristics of the study population at the beginning of each testing session.

	Calf assessments			Forearm assessments		
	Day one	Day seven	p-value	Day one	Day seven	p-value
Fasting blood glucose (g/L)	0.96 ± 0.07	0.95 ± 0.09	0.82	0.95 ± 0.07	0.93 ± 0.08	0.01
Resting heart rate (beats/minute)	68.40 ± 8.85	66.00 ± 4.11	0.33	67.90 ± 10.10	67.30 ± 8.63	0.76
SBP (mmHg)	121.00 ± 8.02	119.00 ± 7.50	0.48	119.00 ± 7.56	119.00 ± 11	0.89
DBP (mmHg)	70.80 ± 4.73	71.60 ± 8.85	0.73	71.80 ± 6.85	71.50 ± 4.67	0.86
MAP (mmHg)	87.40 ± 4.88	87.40 ± 7.39	0.99	87.60 ± 6.00	87.30 ± 6.33	0.86

SBP denotes systolic blood pressure; DBP, diastolic blood pressure; MAP, mean arterial pressure.

### Microvascular responses to each protocol of current-induced vasodilation

When two tests were conducted on the same site in a single testing session, on either the calf or the forearm, the current-induced vasodilation during the first assessment was consistently higher than that of the second assessment conducted an hour later (Table 3). The microvascular response to each protocol conducted on the forearm was also consistently higher than that on the calf (Table 4).

**Table 3:** The intrasite, intraday reproducibility of each protocol used to mediate a current-induced vasodilation when assessed with laser speckle contrast imaging on the calf and the forearm.

	Right calf T1	Right calf T2	CV (%)	ICC [95% CI]	Right forearm T1	Right forearm T2	CV (%)	ICC [95% CI]
**Protocol A**								
Baseline (PU)	30.26 ± 4.85	27.41 ± 4.98	8.60	0.80 [0.37–0.93]	31.41 ± 3.97	28.85 ± 6.11	11.12	0.73 [0.29–0.89]
Baseline (CVC)	0.35 ± 0.07	0.32 ± 0.06	10.14	0.80 [0.37–0.93]	0.38 ± 0.06	0.34 ± 0.08	12.19	0.77 [0.28–0.92]
Plateau (PU)	74.39 ± 19.82	55.08 ± 14.33	27.85	0.21 [-0.35–0.62]	104.65 ± 33.32	78.54 ± 23.47	43.28	-0.05 [-0.89–0.51]
Plateau (CVC)	0.87 ± 0.22	0.64 ± 0.16	28.21	0.24 [-0.32–0.63]	1.25 ± 0.35	0.94 ± 0.30	42.87	0.43 [-0.68–0.54]
Δ (PU)	44.12 ± 18.58	27.66 ± 14.08	79.15	0.13 [-0.51–0.58]	73.24 ± 34.39	49.70 ± 26.06	117.60	0.11 [-0.72–0.60]
Δ (CVC)	0.51 ± 0.21	0.32 ± 0.16	79.81	0.17 [-0.45–0.60]	0.87 ± 0.38	0.60 ± 0.31	116.26	0.22 [-0.52–0.65]
%Δ	149.22 ± 64.39	104.99 ± 59.68	82.04	0.28 [-0.48–0.68]	240.81 ± 115.59	189.20 ± 116.85	121.86	0.42 [-0.35–0.76]
AUC	18619.88 ± 4643.97	17454.16 ± 5937.38	20.25	0.29 [-0.28–0.67]	23260.03 ± 5720.80	17302.93 ± 4219.71	25.50	0.32 [-0.26–0.69]
**Protocol B**								
Baseline (PU)	29.97 ± 5.23	27.86 ± 5.03	10.36	0.77 [0.41–0.91]	30.26 ± 4.80	28.72 ± 5.99	11.98	0.74 [0.36–0.89]
Baseline (CVC)	0.35 ± 0.06	0.33 ± 0.06	11.61	0.74 [0.36–0.89]	0.37 ± 0.06	0.34 ± 0.07	12.96	0.71 [0.30–0.86]
Plateau (PU)	69.62 ± 22.49	61.56 ± 19.53	29.58	0.55 [-0.08–0.82]	87.63 ± 27.95	74.54 ± 25.92	29.59	0.69 [0.23–0.88]
Plateau (CVC)	0.81 ± 0.27	0.72 ± 0.23	30.21	0.60 [0.03–0.84]	1.05 ± 0.33	0.89 ± 0.34	28.37	0.76 [0.36–0.91]
Δ (PU)	39.66 ± 19.51	33.70 ± 16.81	71.99	0.48 [-0.27–0.79]	57.37 ± 27.52	45.82 ± 27.55	93.30	0.71 [0.29–0.88]
Δ (CVC)	0.46 ± 0.24	0.40 ± 0.20	72.88	0.54 [-0.13–0.81]	0.69 ± 0.33	0.56 ± 0.34	92.19	0.77 [0.41–0.91]
%Δ	131.33 ± 60.96	120.26 ± 56.84	66.43	0.53 [-0.20–0.81]	194.03 ± 96.67	173.57 ± 110.89	95.54	0.75 [0.37–0.90]
AUC	17454.16 ± 5937.38	15020.00 ± 3622.68	23.95	0.54 [-0.07–0.81]	19368.46 ± 6300.36	16642.18 ± 4755.76	21.93	0.71 [0.26–0.88]
**Protocol C**								
Baseline (PU)	28.84 ± 3.08	27.81 ± 4.82	9.16	0.79 [0.49–0.92]	32.32 ± 5.11	29.38 ± 6.02	15.24	0.50 [-0,15–0.79]
Baseline (CVC)	0.34 ± 0.04	0.33 ± 0.06	10.17	0.81 [0.53–0.92]	0.39 ± 0.07	0.35 ± 0.08	15.67	0.64 [0.11–0.86]
Plateau (PU)	63.78 ± 21.19	52.72 ± 17.76	20.47	0. 75 [0.25–0.91]	103.60 ± 28.14	80.17 ± 25.46	33.59	0.41 [-0.22–0.75]
Plateau (CVC)	0.74 ± 0.25	0.62 ± 0.20	20.78	0.74 [0.25–0.90]	1.24 ± 0.30	0.96 ± 0.33	34.23	0.48 [-0.17–0.79]
Δ (PU)	34.93 ± 20.53	24.91 ± 16.05	69.66	0. 75 [0.28–0.91]	71.28 ± 28.53	50.80 ± 26.76	88.95	0.51 [-0.11–0.80]
Δ (CVC)	0.41 ± 0.24	0.29 ± 0.18	70.10	0.74 [0.27–0.90]	0.84 ± 0.32	0.61 ± 0.33	90.58	0.57 [-0.04–0.83]
%Δ	121.34 ± 71.55	89.89 ± 56.59	70.70	0.76 [0.34–0.91]	228.13 ± 101.57	185.32 ± 108.01	91.14	0.65 [0.15–0.86]
AUC	16364.50 ± 4629.45	13875.57 ± 3559.92	17.31	0.68 [0.15–0.88]	22965.99 ± 6620.98	17319.89 ± 5079.09	26.10	0.49 [-0.18–0.79]

Intrasite, intraday reproducibility was evaluated for both the calf and forearm by comparing assessment one (T1) on the right limb to assessment two (T2) on the same site of the right limb. The cutaneous microvascular responses to each protocol are presented as the mean ± standard deviation and are reported as the maximal plateau in cutaneous blood perfusion and the change (Δ) in blood perfusion between peak and baseline values, expressed in perfusion units (PU) and cutaneous vascular conductance (CVC); and the percentage change (%Δ) from baseline measurements and the area under the curve (AUC). Coefficients of variation (CV) <35% were deemed acceptable and intra-class correlation coefficient (ICC) values <0.40, 0.40 to 0.75, and >0.75 represented poor, fair to good, and excellent agreements, respectively. CI denotes confidence interval.

**Table 4:** The intersite, intraday reproducibility of each protocol used to mediate a current-induced vasodilation when assessed with laser speckle contrast imaging on the calf and the forearm.

	Right calf T1	Left calf T1	CV (%)	ICC [95% CI]	Right forearm T1	Left forearm T1	CV (%)	ICC [95% CI]
**Protocol A**								
Baseline (PU)	30.26 ± 4.85	27.96 ± 3.60	11.77	0.56 [-0.20–0.82]	31.41 ± 3.97	29.38 ± 5.28	16.61	0.20 [-0.87–0.67]
Baseline (CVC)	0.35 ± 0.07	0.33 ± 0.05	12.58	0.63 [0.13–0.85]	0.38 ± 0.06	0.34 ± 0.07	19.14	0.38 [-0.38–0.74]
Plateau (PU)	74.39 ± 19.82	65.38 ± 20.07	37.22	-0.20 [-1.86–0.51]	104.65 ± 33.32	95.13 ± 21.34	27.72	0.36 [-0.57–0.74]
Plateau (CVC)	0.87 ± 0.22	0.77 ± 0.26	38.25	-0.24 [-2.10–0.51]	1.25 ± 0.35	1.11 ± 0.26	28.72	0.22 [-0.82–0.68]
Δ (PU)	44.12 ± 18.58	37.41 ± 19.17	88.68	-0.19 [-1.98–0.53]	73.24 ± 34.39	65.74 ± 23.18	59.70	0.45 [-0.39–0.78]
Δ (CVC)	0.51 ± 0.21	0.44 ± 0.24	90.05	-0.27 [-2.30–0.50]	0.87 ± 0.38	0.77 ± 0.28	60.14	0.44[-0.39–0.78]
%Δ	149.22 ± 64.39	134.88 ± 69.51	83.19	0.20 [-1.07–0.69]	240.81 ± 115.59	238.10 ± 105.28	64.43	0.55 [-0.17–0.83]
AUC	18619.88 ± 4643.97	16503.54 ± 4469.40	29.05	0.09 [-1.11–0.63]	23260.03 ± 5720.80	22520.78 ± 6072.65	19.65	0.70 [0.23–0.88]
**Protocol B**								
Baseline (PU)	29.97 ± 5.23	27.09 ± 4.84	15.30	0.36 [-0.41–0.73]	30.26 ± 4.80	29.24 ± 4.68	13.36	0.51 [-0.23–0.81]
Baseline (CVC)	0.35 ± 0.06	0.32 ± 0.06	17.02	0.28 [-0.65–0.70]	0.37 ± 0.06	0.34 ± 0.06	16.40	0.30 [-0.67–0.72]
Plateau (PU)	69.62 ± 22.49	59.04 ± 23.14	36.71	0.47 [-0.24–0.78]	87.63 ± 27.95	84.81 ± 29.46	24.86	0.82 [0.55–0.93]
Plateau (CVC)	0.81 ± 0.27	0.70 ± 0.29	36.47	0.56 [-0.05–0.82]	1.05 ± 0.33	0.98 ± 0.32	26.05	0.74 [0.35–0.90]
Δ (PU)	39.66 ± 19.51	31.96 ± 20.98	79.56	0.51 [-0.18–0.80]	57.37 ± 27.52	55.57 ± 30.01	68.68	0.83 [0.58–0.94]
Δ (CVC)	0.46 ± 0.24	0.38 ± 0.26	78.65	0.58 [-0.01–0.83]	0.69 ± 0.33	0.64 ± 0.34	69.01	0.80 [0.49–0.92]
%Δ	131.33 ± 60.96	116.43 ± 74.07	73.35	0.48 [-0.31–0.80]	194.03 ± 96.67	197.53 ± 114.15	70.47	0.77 [0.40–0.91]
AUC	17454.16 ± 5937.38	15567.53 ± 5732.50	31.15	0.57 [-0.05–0.83]	19368.46 ± 6300.36	19745.62 ± 6440.16	15.98	0.91 [0.76–0.96]
**Protocol C**								
Baseline (PU)	28.84 ± 3.08	26.39 ± 4.30	11.45	0.39 [-0.31–0.74]	32.32 ± 5.11	30.24 ± 4.53	15.94	0.22 [-0.84–0.68]
Baseline (CVC)	0.34 ± 0.04	0.31 ± 0.06	12.90	0.55 [-0.05–0.82]	0.39 ± 0.07	0.36 ± 0.07	19.03	0.41 [-0.33–0.76]
Plateau (PU)	63.78 ± 21.19	55.70 ± 17.64	35.25	0.39 [-0.45–0.75]	103.60 ± 28.14	96.55 ± 24.37	26.05	0.72 [0.31–0.89]
Plateau (CVC)	0.74 ± 0.25	0.66 ± 0.22	34.83	0.47 [-0.30–0.78]	1.24 ± 0.30	1.13 ±0.29	27.13	0.61 [0.07–0.84]
Δ (PU)	34.93 ± 20.53	29.31 ± 17.75	145.03	0.43 [-0.41–0.77]	71.28 ± 28.53	66.31 ± 26.02	56.99	0.81 [0.54–0.93]
Δ (CVC)	0.41 ± 0.24	0.35 ± 0.22	144.25	0.46 [-0.35–0.79]	0.84 ± 0.32	0.77 ± 0.30	57.37	0.78 [0.47–0.91]
%Δ	121.34 ± 71.55	114.36 ± 68.12	148.02	0.50 [-0.31–0.80]	228.13 ± 101.57	229.79 ± 105.18	54.01	0.85 [0.61–0.94]
AUC	16364.50 ± 4629.45	14029.68 ± 3700.62	23.11	0.53 [-0.08–0.80]	22965.99 ± 6620.98	21164.36 ± 5504.73	17.24	0.82 [0.54–0.93]

Intersite, intraday reproducibility was evaluated for both the calf and forearm by comparing assessment one (T1) on the right limb to T1 on the left limb. The cutaneous microvascular responses to each protocol are presented as the mean ± standard deviation and are reported as the maximal plateau in cutaneous blood perfusion and the change (Δ) in blood perfusion between peak and baseline values, expressed in perfusion units (PU) and cutaneous vascular conductance (CVC); and the percentage change (%Δ) from baseline measurements and the area under the curve (AUC). Coefficients of variation (CV) <35% were deemed acceptable and intra-class correlation coefficient (ICC) values <0.40, 0.40 to 0.75, and >0.75 represented poor, fair to good, and excellent agreements, respectively. CI denotes confidence interval; T1, assessment one.

Considering the typical microvascular response to each protocol of current-induced vasodilation across the entire study population, any assessment where the increase from baseline values was <10 perfusion units was defined as a ‘no response’ case. In 180 assessments on the calf across the entire study population, there were 21 assessments where there wasn’t a microvascular response to the electrical stimulation (mean increase from baseline values of 5.05 ± 1.93 perfusion units). In 180 assessments on the forearm, there were 16 cases (6.86 ± 2.52 perfusion units).

No response cases were sporadic. A microvascular response may have been induced in a participant during the first assessment, but not the second assessment in intrasite testing, and vice-versa. Similarly, a microvascular response may have been induced on the right limb, but not the left limb in intersite testing, and vice-versa. There were also instances where vasodilation wasn’t induced by one protocol, but it was induced by the other protocols being tested simultaneously. Further, no protocol was more prone to no response cases than any other. While no responses cases were sporadic, they were limited to a few participants.

### Data expression affects reproducibility

For all protocols on both the calf and forearm, intraday (intrasite and intersite) and interday reproducibility of laser speckle contrast imaging data was consistently unacceptable when the microvascular response to the electrical stimulation was expressed as the change between baseline values and the maximal plateau in perfusion units or as cutaneous vascular conductance, and as the percentage increase from baseline to the maximal plateau (Tables 3–5). The reproducibility of each protocol improved substantially when the microvascular response was reported as the maximal plateau in perfusion units or as cutaneous vascular conductance, and as the area under the curve.

**Table 5:** The interday reproducibility of each protocol used to mediate a current-induced vasodilation when assessed with laser speckle contrast imaging on the calf and the forearm.

	Calf assessments				Forearm assessments			
	Day one	Day seven	CV (%)	ICC [95% CI]	Day one	Day seven	CV (%)	ICC [95% CI]
**Protocol A**								
Baseline (PU)	29.70 ± 3.81	28.53 ± 4.90	9.39	0.80 [0.51–0.92]	30.22 ± 4.58	30.58 ± 4.97	10.35	0.76 [0.38–0.91]
Baseline (CVC)	0.35 ± 0.06	0.33 ± 0.06	9.17	0.84 [0.59–0.94]	0.36 ± 0.06	0.37 ± 0.07	13.91	0.74 [0.32–0.90]
Plateau (PU)	72.86 ± 18.73	66.90 ± 21.66	30.29	0.58 [-0.03–0.83]	103.88 ± 23.44	95.90 ± 32.10	21.55	0.84 [0.61–0.94]
Plateau (CVC)	0.86 ± 0.23	0.78 ± 0.25	32.26	0.51 [-0.19–0.80]	1.23 ± 0.25	1.13 ± 0.36	22.12	0.77 [0.44–0.91]
Δ (PU)	43.17 ± 17.74	38.37 ± 20.24	96.55	0.51 [-0.23–0.81]	73.67 ± 23.74	65.31 ± 33.90	58.27	0.83 [0.58–0.93]
Δ (CVC)	0.51 ± 0.21	0.45 ± 0.24	99.25	0.44 [-0.40–0.78]	0.87 ± 0.26	0.76 ± 0.39	58.58	0.78 [0.47–0.91]
%Δ	146.91 ± 61.08	137.19 ± 72.83	99.68	0.40 [-0.58–0.76]	251.30 ± 86.53	227.61 ± 129.10	64.52	0.78 [0.47–0.92]
AUC	18086.62 ± 4310.52	17279.49 ± 6221.00	21.61	0.78 [0.47–0.91]	23517.95 ± 5638.68	22262.86 ± 6104.79	15.42	0.86 [0.66–0.95]
**Protocol B**								
Baseline (PU)	29.17 ± 4.66	27.89 ± 5.71	14.44	0.59 [-0.01–0.84]	29.26 ± 3.99	30.24 ± 5.40	12.29	0.60 [-0.01–0.84]
Baseline (CVC)	0.34 ± 0.06	0.32 ± 0.06	14.55	0.56 [-0.07–0.82]	0.35 ± 0.04	0.36 ± 0.07	15.61	0.39 [-0.57–0.76]
Plateau (PU)	66.90 ± 24.38	61.77 ± 22.18	34.07	0.63 [0.06–0.85]	87.55 ± 25.52	84.89 ± 31.59	23.08	0.80 [0.48–0.92]
Plateau (CVC)	0.79 ± 0.31	0.72 ± 0.25	35.42	0.56 [-0.09–0.83]	1.04 ± 0.29	1.00 ± 0.36	24.38	0.72 [0.28–0.89]
Δ (PU)	37.73 ± 21.53	33.89 ± 19.52	86.66	0.59 [-0.05–0.84]	58.29 ± 26.26	54.65 ± 31.04	61.46	0.81 [0.52–0.93]
Δ (CVC)	0.45 ± 0.27	0.39 ± 0.23	88.86	0.52 [-0.20–0.81]	0.69 ± 0.31	0.64 ± 0.36	62.10	0.77 [0.43–0.91]
%Δ	126.22 ± 66.04	121.54 ± 70.34	81.39	0.52 [-0.25–0.81]	205.64 ± 98.92	185.93 ± 111.32	66.92	0.81 [0.54–0.93]
AUC	17279.49 ± 6221.00	15742.20 ± 5481.13	26.90	0.71 [0.29–0.89]	19593.03 ± 5914.90	19521.05 ± 6801.21	15.84	0.87 [0.67–0.95]
**Protocol C**								
Baseline (PU)	28.48 ± 4.13	26.76 ± 3.54	7.94	0.78 [0.41–0.92]	30.99 ± 4.57	31.57 ± 5.28	15.88	0.20 [-1.14–0.69]
Baseline (CVC)	0.34 ± 0.06	0.31 ± 0.05	9.28	0.78 [0.41–0.92]	0.37 ± 0.07	0.38 ± 0.08	19.14	0.39 [-0.61–0.76]
Plateau (PU)	60.77 ± 21.69	58.70 ± 17.94	30.88	0.72 [0.28–0.89]	100.05 ± 25.37	100.10 ± 27.71	21.01	0.85 [0.62–0.94]
Plateau (CVC)	0.71 ± 0.26	0.69 ± 0.22	32.84	0.66 [0.11–0.86]	1.19 ± 0.29	1.18 ± 0.31	22.39	0.80 [0.48–0.92]
Δ (PU)	32.30 ± 20.85	31.95 ± 17.84	171.20	0.66 [0.11–0.87]	69.06 ± 26.66	68.53 ± 28.17	52.20	0.88 [0.69–0.95]
Δ (CVC)	0.38 ± 0.25	0.37 ± 0.21	173.77	0.62 [0.01–0.85]	0.82 ± 0.31	0.80 ± 0.32	52.97	0.87 [0.66–0.95]
%Δ	113.73 ± 70.89	121.97 ± 68.73	178.01	0.57 [-0.12–0.83]	232.27 ± 105.48	225.65 ± 101.16	57.18	0.86 [0.64–0.94]
AUC	15511.00 ± 4916.74	14883.17 ± 3689.69	18.85	0.78 [0.44–0.91]	22523.97 ± 6038.35	21606.38 ± 6240.43	15.76	0.91 [0.77–0.96]

Interday reproducibility was evaluated for both the calf and forearm by comparing assessment one (T1) on both the right and left limbs at day one to T1 on both the right and left limbs at day seven. The cutaneous microvascular responses to each protocol are presented as the mean ± standard deviation and are reported as the maximal plateau in cutaneous blood perfusion and the change (Δ) in blood perfusion between peak and baseline values, expressed in perfusion units (PU) and cutaneous vascular conductance (CVC); and the percentage change (%Δ) from baseline measurements and the area under the curve (AUC). Coefficients of variation (CV) <35% were deemed acceptable and intra-class correlation coefficient (ICC) values <0.40, 0.40 to 0.75, and >0.75 represented poor, fair to good, and excellent agreements, respectively. CI denotes confidence interval.

### Intraday (intrasite and intersite) and interday reproducibility

All data, including no response cases, were pooled in the primary analysis. Considering the effect data expression has on reproducibility, the following interpretations are based on data expressed as the maximal plateau in perfusion units or as cutaneous vascular conductance, and as the area under the curve.

Overall, the intrasite, intraday reproducibility of Protocol A (Pulses of 0.03, 0.06, 0.09, 0.12, 0.15, and 0.18 mA for 10 s each; 60 s intervals) could be summarized as poor or unclear at best (Table 3). In comparison, the intrasite reproducibility of Protocol B (0.1 mA for 60 s) was far better, rated good to excellent on the forearm. When same-site assessments were conducted on the calf, Protocol B was slightly less reproducible, but still rated good. Protocol C (2 pulses of 0.1 mA for 10s each; 240 s interval) had good to excellent intrasite reproducibility in assessments on the calf, but only fair reproducibility on the forearm.

In general, the intraday reproducibility of Protocol A didn’t improve by changing the site for the second assessment of the testing session (Table 4). Similarly, the intersite reproducibility of Protocol B was consistent with its intrasite reproducibility in assessments on the forearm (good to excellent), but its reproducibility deteriorated in assessments on the calf. The intraday reproducibility of Protocol C declined substantially with intersite assessments on the calf. While, in contrast, reproducibility substantially improved with intersite assessments on the forearm.

The interday reproducibility of each protocol was excellent when assessments were performed on the forearm (Table 5). Assessments on the calf were less reproducible when performed at a seven-day interval. However, they were still rated good overall.

### Sensitivity analysis excluding no response cases

There were many instances where highly unacceptable coefficients of variation were reported next to good or excellent intra-class correlation coefficients. To determine what was driving these conflicting findings, a sensitivity analysis was performed where comparisons that included a no response case (i.e., where the participant didn’t have a microvascular response to the electrical stimulation) were removed from the analysis. It is important to note that even with the removal of comparisons with no-response cases, there were still enough comparisons to achieve statistical power. While in many instances the intra-class correlation coefficients were more in agreeance with the coefficients of variation, some conflicting findings remained (S1 and S2 Tables). The interday reproducibility of each protocol were consistent with or improved from those of the primary analysis (S3 Table), good to excellent on both the calf and forearm.

## Discussion

In evaluations of three protocols for mediating a current-induced vasodilation, this study found that the test is most reproducible when performed at a seven-day interval and when data are expressed as the maximal plateau in perfusion units or as cutaneous vascular conductance, and as the area under the curve. The intrasite, intraday reproducibility of each protocol was less clear, with two out three of the protocols deemed acceptable and reproducibility that varied depending on anatomical site being assessed. Intersite testing didn’t improve intraday reproducibility consistently across the protocols. It must also be noted that a microvascular response wasn’t induced by the electrical stimulation in ∼10% of assessments on either the calf or forearm.

Given the difference in the clarity of the results between intraday and interday testing, this study might simply recommend that any future research using current-induced vasodilation perform interday assessments to best isolate the effect of the intervention being studied. This doesn’t preclude intraday assessments from being incorporated in a study design. However, more consideration must be given to which protocol is chosen depending on which anatomical site is being assessed. A set of recommendations based on the findings from this present study is detailed in Table 6, summarizing in which study designs each protocol is suitable.

**Table 6:** Recommendations for using current-induced vasodilation based on the findings from this present study.

	CALF	FOREARM
**Intrasite, intraday assessments**	Two pulses of 0.1 mA for 10 s each; 240 s interval **Good to excellent reproducibility**	0.1 mA for 60 s **Good to excellent reproducibility**
**Intersite, intraday assessments**	0.1 mA for 60 s **Good** **OR** Two pulses of 0.1 mA for 10 s each; 240 s interval **Good reproducibility**	0.1 mA for 60 s **Good to Excellent** **OR** Two pulses of 0.1 mA for 10 s each; 240 s interval **Good to excellent reproducibility**
**Interday assessments**	Six pulses of 0.03, 0.06, 0.09, 0.12, 0.15, and 0.18 mA for 10 s each; 60 s intervals **Good reproducibility** **OR** 0.1 mA for 60 s **Good reproducibility** **OR** Two pulses of 0.1 mA for 10 s each; 240 s interval **Good reproducibility**	Six pulses of 0.03, 0.06, 0.09, 0.12, 0.15, and 0.18 mA for 10 s each; 60 s intervals **Excellent** **OR** 0.1 mA for 60 s **Good to excellent reproducibility** **OR** Two pulses of 0.1 mA for 10 s each; 240 s interval **Excellent reproducibility**

It is recommended that data are expressed as the maximal plateau in perfusion units or as cutaneous vascular conductance, and as the area under the curve.

The inconsistencies in intraday reproducibility between each protocol suggest that researchers adopt these recommendations with caution. Indeed, it is encouraged that future research utilizing current-induced vasodilation still assess and report the reproducibility of its chosen protocol in its study population, whether intraday or interday assessments are being performed. Given that a microvascular response wasn’t induced in ∼10% of assessments on the calf or forearm, it is also recommended that future studies test their chosen protocol in their study population to ensure that a potential participant is a consistent responder to the electrical provocation before enrolling them in a demanding study protocol. Indeed, the findings from this present study suggest that some persons may be categorized as non-responders to protocols of current-induced vasodilation. Although, it isn’t clear as to why.

Consistent with previous reproducibility studies used laser speckle contrast imaging to quantify a microvascular response [10, 12, 18, 19], this study’s results indicate that reproducibility is best when data are reported as the maximal plateau in perfusion units or as cutaneous vascular conductance. In contrast to those previous studies, this study also indicates that data are highly reproducible when expressed as the area under the curve. This improvement in the reproducibility of data expressed as area under the curve may be explained in part by less variance, than that seen in previous research, in baseline measurements of blood perfusion between tests. Further contradicting previous research, this study indicates that data shouldn’t be expressed as the change between baseline values and the maximal plateau in the microvascular response to the electrical stimulation.

### Study limitations

It must be noted that fasting blood glucose concentrations were different between day one and day seven assessments on the forearm. It is not known to what extent this difference affected interday reproducibility. However, given that reproducibility was still good to excellent, its impact was deemed negligible.

Although we regularly measured blood pressure several times within each visit, we cannot rule out variations in pressure during the one-hour measurement period. However, we observed that results were very consistent between arbitrary perfusion units and cutaneous vascular conductance, which suggests a relative stability in pressure. This is likely a result of the strict acclimatization period and room temperature control, and a calm environment during visits.

While we didn’t formally compare T1 vs T2, we observed a generally lower value for T2 than for T1. This is unlikely to be due to insufficient recovery time, since baseline is also affected. This could be due to a change in skin temperature, although previous work by our group rather suggests an opposite evolution over time, i.e., a modest time-dependent increase in perfusion during the day [20]. However, we didn’t control for skin temperature, while some authors have standardized skin temperature to limit baseline variability [21]. We have previously shown that it slightly improves reproducibility of post-occlusive hyperemia measured with laser Doppler, but that its impact is modest compared to the improvement in reproducibility brought by the use of laser speckle contrast imaging, the technique that we have used in the present work [12]. In addition, standardizing skin temperature while performing current-induced vasodilation would require custom-made probes, which would decrease the external validity of this work. This is relevant issue when expressing data as a variation from baseline.

In the present study, reproducibility is worse when data are expressed a percent change from baseline. This is consistent with a previous study, which explored the reproducibility of local thermal hyperemia with laser speckle contrast imaging [12]. Interestingly, when data were expressed as the area under the curve, which also considers the baseline value, reproducibility was much better. Although we do not have a clear explanation about this phenomenon, we hypothesize that reproducibility is dramatically impaired when expressing data as a percent change from baseline because of the variability of baseline flux and that of the plateau potentiate; while expressing data as area under the curve integrates both the magnitude and the length of the vasodilation, which might smooth the overall response and lessen the importance of baseline flow.

We didn’t account for the period of the hormonal cycle that the women participants were, which may introduce additional heterogeneity. Indeed, sex hormones may have an influence on endothelium-dependent skin microvascular function, although probably limited [2]. Other endothelium-dependent reactivity tests, namely post-occlusive and thermal hyperemia plateau, measured in healthy young women were not affected by a treatment with a gonadotropin-releasing hormone antagonist, which suppresses endogenous sex hormones. However, the initial peak following local heating was affected [22]. In the light of these data, we checked whether non-response was associated with contraceptive use and found no obvious association. Yet the question of the impact of estrogens and progesterone on current-induced vasodilation remains and deserves further attention.

The interpretation of the results was difficult in some instances where highly unacceptable coefficients of variation were reported next to good or excellent intra-class correlation coefficients. Sensitivity analyses suggest that these conflicting findings were mostly driven by analysis of a heterogeneous population, as most of the discrepancies were corrected by removal of non-responders from the analysis of interday correaltions. While it was assumed that a homogenous, healthy population was recruited to participate in the study, there were two subgroups consisting of those who always had a microvascular response to the electrical stimulation and those who didn’t. Analysis of intra-class correlation coefficients on a heterogeneous population, produces artificially high intra-class correlation coefficients not representative of the true reproducibility [23].

Although Protocol A (Pulses of 0.03, 0.06, 0.09, 0.12, 0.15, and 0.18 mA for 10 s each; 60 s intervals) mediates a current-induced vasodilation, further research is needed to verify the underlying pathways of the subsequent microvascular response. In the context of further research, additional studies are also required identify/develop the single, ‘best’ protocol for mediating a current-induced vasodilation to study the PGI_2_ pathway. This present study can’t do that and, thus, several protocols are recommended. Indeed, refinement of the technique is necessary to ensure the universal adoption of a single protocol, which will aid in comparisons between future research and contribute to the development of a broader consensus in microvascular research.

### Conclusion

This study indicates that current-induced vasodilation is most reproducible when interday assessments are conducted and when data are expressed as the maximal plateau in perfusion units or as cutaneous vascular conductance, and as the area under the curve. Due to the sporadic no response cases and the inconsistencies in intraday reproducibility between protocols, current-induced vasodilation should be used with caution.

## Supporting information

S1 Table The intrasite, intraday reproducibility of each protocol used to mediate a current-induced vasodilation when assessed with laser speckle contrast imaging on the calf and the forearm. (DOCX)

S2 Table The intersite, intraday reproducibility of each protocol used to mediate a current-induced vasodilation when assessed with laser speckle contrast imaging on the calf and the forearm. (DOCX)

S3 Table The interday reproducibility of each protocol used to mediate a current-induced vasodilation when assessed with laser speckle contrast imaging on the calf and the forearm. (DOCX)
