# Supplementary material for: The reproducibility of protocols used to mediate a current-induced vasodilation in the human cutaneous microcirculation
Source: PLoS One. 2024 Nov 27;19(11):e0314430. doi: 10.1371/journal.pone.0314430 (PMC11602075; doi:10.1371/journal.pone.0314430)
Supplement: S1 Table — (DOCX) [file pone.0314430.s001.docx]

**S1 Table.** The intrasite, intraday reproducibility of each protocol used to mediate a current-induced vasodilation when assessed with laser speckle contrast imaging on the calf and the forearm

|  | **Right calf T1** | **Right calf T2** | **CV (%)** | **ICC [95% CI]** | **Right forearm T1** | **Right forearm T2** | **CV (%)** | **ICC [95% CI]** |
| --- | --- | --- | --- | --- | --- | --- | --- | --- |
| **Protocol A** |  |  |  |  |  |  |  |  |
| Plateau (PU) | 75.85 ± 18.36 | 58.28 ± 13.02 | 23.39 | 0.22 [-0.40 - 0.65] | 105.00 ± 18.76 | 89.38 ± 14.72 | 19.33 | 0.30 [-0.44 - 0.72] |
| Plateau (CVC) | 0.88 ± 0.21 | 0.68 ± 0.15 | 22.55 | 0.29 [-0.33 - 0.69] | 1.28 ± 0.22 | 1.08 ± 0.17 | 19.27 | 0.22 [-0.48 - 0.67] |
| Δ (PU) | 45.71 ± 16.73 | 30.88 ± 12.57 | 45.38 | 0.10 [-0.62 - 0.60] | 73.96 ± 21.15 | 61.67 ± 16.43 | 36.88 | 0.59 [-0.08 - 0.86] |
| Δ (CVC) | 0.53 ± 0.19 | 0.36 ± 0.14 | 43.70 | 0.17 [-0.50 - 0.63] | 0.90 ± 0.25 | 0.74 ± 0.19 | 36.36 | 0.54 [-0.16 - 0.84] |
| %Δ | 155.61 ± 59.09 | 117.30 ± 55.58 | 47.20 | 0.27 [-0.63 - 0.71] | 247.17 ± 83.94 | 236.55 ± 90.89 | 33.96 | 0.83 [0.48 - 0.94] |
| AUC | 19181.76 ± 4644.25 | 18436.16 ± 5896.44 | 20.55 | 0.21 [-0.39 - 0.64] | 24450.33 ± 5009.53 | 18589.59 ± 3991.73 | 20.49 | 0.33 [-0.29 - 0.73] |
| **Protocol B** |  |  |  |  |  |  |  |  |
| Plateau (PU) | 71.66 ± 22.07 | 64.83 ± 17.61 | 26.41 | 0.49 [-0.30 - 0.81] | 96.10 ± 22.48 | 83.03 ± 21.28 | 27.91 | 0.43 [-0.36 - 0.79] |
| Plateau (CVC) | 0.84 ± 0.27 | 0.76 ± 0.21 | 26.28 | 0.55 [-0.16 - 0.83] | 1.16 ± 0.26 | 1.00 ± 0.28 | 25.75 | 0.58 [-0.08 - 0.85] |
| Δ (PU) | 41.63 ± 18.99 | 36.65 ± 14.89 | 50.92 | 0.36 [-0.70 - 0.76] | 66.42 ± 21.05 | 55.75 ± 20.84 | 61.51 | 0.32 [-0.71 - 0.75] |
| Δ (CVC) | 0.49 ± 0.23 | 0.43 ± 0.17 | 50.41 | 0.44 [-0.49 - 0.79] | 0.80 ± 0.25 | 0.68 ± 0.26 | 58.24 | 0.47 [-0.34 - 0.81] |
| %Δ | 138.11 ± 58.88 | 130.52 ± 49.71 | 49.20 | 0.40 [-0.68 - 0.78] | 226.80 ± 73.62 | 212.67 ± 86.12 | 68.11 | 0.35 [-0.95 - 0.78] |
| AUC | 18037.05 ± 5965.74 | 15583.40 ± 3330.81 | 24.36 | 0.46 [-0.29 - 0.79] | 20992.86 ± 5885.07 | 17440.46 ± 4925.69 | 20.66 | 0.65 [0.03 - 0.88] |
| **Protocol C** |  |  |  |  |  |  |  |  |
| Plateau (PU) | 71.68 ± 16.78 | 59.40 ± 15.23 | 20.84 | 0.54 [-0.17 - 0.84] | 110.00 ± 21.70 | 87.03 ± 20.62 | 27.81 | 0.10 [-0.56 - 0.58] |
| Plateau (CVC) | 0.84 ± 0.19 | 0.70 ± 0.17 | 20.71 | 0.48 [-0.24 - 0.81] | 1.31 ± 0.22 | 1.04 ± 0.27 | 27.75 | 0.20 [-0.42 - 0.64] |
| Δ (PU) | 42.71 ± 16.21 | 31.21 ± 13.38 | 40.27 | 0.52 [-0.19 - 0.83] | 78.02 ± 22.33 | 58.64 ± 20.34 | 59.77 | 0.09 [-0.73 - 0.60] |
| Δ (CVC) | 0.50 ± 0.19 | 0.36 ± 0.15 | 39.49 | 0.48 [-0.25 - 0.81] | 0.93 ± 0.24 | 0.71 ± 0.25 | 59.38 | 0.18 [-0.58 - 0.65] |
| %Δ | 148.21 ± 56.57 | 112.37 ± 46.64 | 40.30 | 0.53 [-0.18 - 0.83] | 251.94 ± 85.76 | 214.95 ± 87.37 | 65.78 | 0.35 [-0.67 - 0.76] |
| AUC | 18068.31 ± 3948.62 | 14970.09 ± 3443.85 | 18.90 | 0.47 [-0.25 - 0.81] | 24536.25 ± 5744.70 | 17930.04 ± 5201.76 | 23.12 | 0.42 [-0.26 - 0.77] |

Intrasite, intraday reproducibility was evaluated for both the calf and forearm by comparing assessment one (T1) on the right limb to assessment two (T2) on the same site of the right limb. The cutaneous microvascular responses to each protocol are presented as the mean ± standard deviation and are reported as the maximal plateau in cutaneous blood perfusion and the change (Δ) in blood perfusion between peak and baseline values, expressed in perfusion units (PU) and cutaneous vascular conductance (CVC); and the percentage change (%Δ) from baseline measurements and the area under the curve (AUC). Coefficients of variation (CV) <35 % were deemed acceptable and intra-class correlation coefficient (ICC) values <0.40, 0.40 to 0.75, and >0.75 represented poor, fair to good, and excellent agreements, respectively. CI denotes confidence interval.
