# Supplementary material for: The reproducibility of protocols used to mediate a current-induced vasodilation in the human cutaneous microcirculation
Source: PLoS One. 2024 Nov 27;19(11):e0314430. doi: 10.1371/journal.pone.0314430 (PMC11602075; doi:10.1371/journal.pone.0314430)
Supplement: S3 Table — (DOCX) [file pone.0314430.s003.docx]

**S3 Table.** The interday reproducibility of each protocol used to mediate a current-induced vasodilation when assessed with laser speckle contrast imaging on the calf and the forearm

|  | **Calf assessments** | |  |  | **Forearm assessments** | |  |  |
| --- | --- | --- | --- | --- | --- | --- | --- | --- |
|  | **Day one** | **Day seven** | **CV (%)** | **ICC [95% CI]** | **Day one** | **Day seven** | **CV (%)** | **ICC [95% CI]** |
| **Protocol A** |  |  |  |  |  |  |  |  |
| Plateau (PU) | 74.40 ± 17.41 | 74.20 ± 17.38 | 17.22 | 0.77 [0.32 - 0.92] | 106.97 ± 20.82 | 102.10 ± 27.29 | 15.74 | 0.87 [0.67 - 0.95] |
| Plateau (CVC) | 0.87 ± 0.21 | 0.86 ± 0.20 | 19.25 | 0.73 [0.21 - 0.91] | 1.25 ± 0.22 | 1.20 ± 0.32 | 16.72 | 0.83 [0.56 - 0.94] |
| Δ (PU) | 44.72 ± 16.17 | 45.39 ± 15.31 | 30.01 | 0.73 [0.21 - 0.91] | 77.20 ± 19.75 | 71.84 ± 28.85 | 38.96 | 0.84 [0.59 - 0.94] |
| Δ (CVC) | 0.52 ± 0.19 | 0.53 ± 0.18 | 32.49 | 0.70 [0.10 - 0.90] | 0.90 ± 0.21 | 0.84 ± 0.33 | 39.61 | 0.82 [0.53 - 0.93] |
| %Δ | 152.59 ± 54.36 | 160.76 ± 56.94 | 30.67 | 0.61 [-0.14 - 0.87] | 264.20 ± 72.88 | 250.72 ± 113.88 | 46.27 | 0.76 [0.36 - 0.91] |
| AUC | 18550.34 ± 4390.14 | 18294.55 ± 6316.75 | 23.12 | 0.86 [0.58 - 0.95] | 24111.80 ± 5440.72 | 23335.98 ± 5424.04 | 12.20 | 0.88 [0.68 -0.95] |
| **Protocol B** |  |  |  |  |  |  |  |  |
| Plateau (PU) | 71.21 ± 23.62 | 67.99 ± 19.06 | 29.30 | 0.58 [-0.23 - 0.86] | 90.41 ± 22.67 | 87.38 ± 30.37 | 23.50 | 0.74 [0.33 - 0.90] |
| Plateau (CVC) | 0.84 ± 0.30 | 0.78 ± 0.23 | 30.03 | 0.55 [-0.30 - 0.84] | 1.07 ± 0.26 | 1.03 ± 0.35 | 24.87 | 0.65 [0.07 - 0.87] |
| Δ (PU) | 42.36 ± 20.56 | 39.25 ± 17.07 | 61.72 | 0.47 [-0.58 - 0.82] | 61.26 ± 23.27 | 56.99 ± 30.02 | 46.73 | 0.76 [0.39 - 0.91] |
| Δ (CVC) | 0.50 ± 0.26 | 0.45 ± 0.20 | 62.45 | 0.41 [-0.74 - 0.80] | 0.72 ± 0.27 | 0.67 ± 0.34 | 47.63 | 0.71 [0.26 - 0.89] |
| %Δ | 143.04 ± 60.73 | 139.91 ± 63.25 | 61.91 | 0.27 [-1.29 - 0.75] | 216.16 ± 89.40 | 193.75 ± 108.57 | 49.99 | 0.78 [0.44 - 0.91] |
| AUC | 18313.90 ± 6295.85 | 17036.39 ± 5201.84 | 25.89 | 0.69 [0.12 - 0.89] | 20073.73 ± 5661.40 | 19998.43 ± 6634.39 | 16.31 | 0.85 [0.61 - 0.94] |
| **Protocol C** |  |  |  |  |  |  |  |  |
| Plateau (PU) | 69.32 ± 18.07 | 65.17 ± 14.18 | 13.95 | 0.81 [0.44 - 0.94] | 105.32 ± 20.50 | 106.15 ± 21.70 | 21.83 | 0.71 [0.20 - 0.89] |
| Plateau (CVC) | 0.81 ± 0.23 | 0.76 ± 0.17 | 17.10 | 0.73 [0.21 - 0.91] | 1.24 ± 0.24 | 1.24 ± 0.25 | 22.98 | 0.63 [-0.02 - 0.86] |
| Δ (PU) | 40.71 ± 16.98 | 38.49 ± 13.18 | 29.96 | 0.74 [0.19 - 0.92] | 74.85 ± 20.76 | 74.66 ± 22.04 | 45.82 | 0.77 [0.38 - 0.92] |
| Δ (CVC) | 0.48 ± 0.20 | 0.45 ± 0.16 | 32.90 | 0.70 [0.05 -0.90] | 0.88 ± 0.24 | 0.87 ± 0.25 | 45.92 | 0.76 [0.33 - 0.91] |
| %Δ | 142.28 ± 56.42 | 146.00 ± 51.97 | 35.06 | 0.63 [-0.23 - 0.88] | 252.63 ± 89.23 | 245.60 ± 84.57 | 46.02 | 0.77 [0.38 - 0.92] |
| AUC | 17318.23 ± 4591.42 | 16170.03 ± 3077.83 | 14.61 | 0.77 [0.31 - 0.92] | 23413.79 ± 5685.26 | 22592.30 ± 5717.79 | 16.13 | 0.88 [0.69 - 0.96] |

Interday reproducibility was evaluated for both the calf and forearm by comparing assessment one (T1) on both the right and left limbs at day one to T1 on both the right and left limbs at day seven. The cutaneous microvascular responses to each protocol are presented as the mean ± standard deviation and are reported as the maximal plateau in cutaneous blood perfusion and the change (Δ) in blood perfusion between peak and baseline values, expressed in perfusion units (PU) and cutaneous vascular conductance (CVC); and the percentage change (%Δ) from baseline measurements and the area under the curve (AUC). Coefficients of variation (CV) <35 % were deemed acceptable and intra-class correlation coefficient (ICC) values <0.40, 0.40 to 0.75, and >0.75 represented poor, fair to good, and excellent agreements, respectively. CI denotes confidence interval.
